# Unintended medication discrepancies across key stages of the in-hospital medication process: a retrospective real-world study in hospitalized patients

**DOI:** 10.1186/s12913-026-14581-4

**Published:** 2026-04-18

**Authors:** Christian Hermann, Ute Amann, Tobias Rüther, Stefan Kääb, Jennifer Nadal, Caroline Rösch, Sofie Baierl, Julian Steinbrech, Maximilian Günther, Dorothea Strobach

**Affiliations:** 1 Hospital Pharmacy, LMU University Hospital, Marchioninistr. 15, 81377 Munich, Germany; 2 Doctoral Program Clinical Pharmacy, LMU University Hospital, Marchioninistr. 15, 81377 Munich, Germany; 3 Faculty of Medicine, LMU, Munich, Germany; 4 Clinic for Psychiatry and Psychotherapy, LMU University Hospital, Munich, Germany; 5 Department of Medicine I, LMU University Hospital, Munich, Germany; 6 German Centre for Cardiovascular Research (DZHK), partner site: Munich Heart Alliance, Munich, Germany; 7 Institute for Medical Biometry, Informatics and Epidemiology, University Hospital Bonn, Bonn, Germany

**Keywords:** Drug therapy safety, Hospital, Unintended medication discrepancies, Medication errors, Medication process

## Abstract

**Background:**

The in-hospital medication process is vulnerable to medication errors, especially during care transitions, when medication discrepancies occur most frequently. However, previous studies focused only on medication errors at single points in this process. This study therefore investigated key stages of the medication process. Also, we examined potential risk factors for unintended medication discrepancies (UMDs) at admission.

**Methods:**

A single-center retrospective study was conducted on seven reference wards from different medical specialties. Technical UMDs at admission, intra-hospital transfer, dispensing, and discharge were evaluated and categorized for patients hospitalized on two predefined days in November 2023. Potential risk factors for UMDs at admission were analyzed using descriptive statistics and logistic regression.

**Results:**

During the study period, 449 UMDs were identified in 215 patients (Median 1 per patient, range 0–15). Of these, 150 (33.4%) were classified in their potential clinical relevance as low severity, 157 (35.0%) as medium, and 142 (31.6%) as severe. The majority of UMDs (206; 45.9%) occurred at admission, of which 104 (50.5%) were still present in discharge letters. Changes in clinical parameters (blood pressure, oxygen saturation, hyper/hypoglycemia) were associated with 37 (18.0%) UMDs for 27 patients at admission. During intra-hospital transfers, 49 UMDs (10.9%) were detected, 4 between general wards and 45 between general and intensive care units. A total of 90 UMDs (20.0%) were identified during dispensing and 104 (23.2%) at discharge.The multivariable logistic regression showed that patients with ≥10 drugs had an increased risk of UMDs at admission (OR 4.0; 95% CI 1.767–9.277; *p* < 0.004). Medication histories taken by pharmacists were associated with reduced UMDs at admission (OR 0.13; 95% CI 0.042–0.414; *p* < 0.001) compared to ward staff.

**Discussion:**

Our findings demonstrate that UMDs are frequent across the hospital pathway, with admission as the most vulnerable point. Taking ≥ 10 drugs was a risk factor, while pharmacist-led medication histories reduced the occurrence of UMDs, highlighting the importance of structured reconciliation. By covering admission, transfers, dispensing, and discharge in a cohort, this study provides a comprehensive perspective on medication process, emphasizing the need for targeted interventions during hospitalization and discharge.

**Supplementary Information:**

The online version contains supplementary material available at 10.1186/s12913-026-14581-4.

## Background

Medication errors are defined as a “deviation from the optimal medication process for the patients that results in or could result in a fundamentally preventable harm” [1]. Medication errors account for one-quarter of all healthcare-related errors and can lead to serious consequences for patients like worsening of medical conditions, hospital admission, prolonged hospital stay, or even death [2]. Their costs amount to around 42 billion USD annually worldwide, which corresponds to nearly 1% of global health expenditure [3]. The medication process consists of multiple steps including medication reconciliation at transition of care, prescribing, dispensing, administration, documentation, monitoring, patient counseling, communication between healthcare facilities, and assessment of therapeutic results. In addition, patient self-administered drugs including complementary and alternative medicine and medication adherence need to be taken into account [1]. Medication errors can occur at any stage of the medication process. One important cause of medication errors are unintended medication discrepancies (UMD) which are defined as unintentional deviations from the intended medication occurring at the various steps of the medication process, e.g. transition of care or in dispensing [4].

The prescribing process can be divided in an intellectual part, e.g. decision for one specific drug and dose based on the diagnosis, and a technical part which includes the appropriate communication of information regarding drug name, dose, form and frequency of administration [5, 6]

UMDs at transitions of care within the healthcare system represent a critical factor contributing to the occurrence of medication errors and adverse drug events [4, 7]. Between 29% and up to 54% of hospitalized patients are affected by at least one discrepancy at admission, most commonly the omission of drugs that the patient normally takes [8–11]. A systematic review has also shown that between 11 and 59% of the identified discrepancies were clinically relevant [12]. However, UMDs can also occur during in-hospital transitions between general wards or from or to the intensive care unit (ICU). A multicenter retrospective point-prevalence study in 985 ICU patients found that 45.7% experienced at least one medication error when being transferred from the ICU to non-ICU care, 75% of which reached the patients [13]. Furthermore, studies in various settings have shown that drug dispensing by nursing staff can lead to dispensing errors as a special kind of UMD in 1.8 to 21.2% of cases, depending on the evaluated dosage form and the documentation system used [14–17]. Previous studies have evaluated UMDs at specific steps of the medication process in hospitals and measures to improve it [18–27]. However, the medication process, including all key steps and the clinical relevance of unintentional medication discrepancies at each stage, has not yet been comprehensively studied. Such an analysis may reveal important opportunities to improve in-hospital medication safety. Prior to the introduction of a unit-dose dispensing system in our hospital we wanted to further evaluate these questions in a comprehensive review of the technical medication process. Therefore, the aim of this study was to investigate, monitor, and evaluate the medication process from hospital admission to discharge in patients from various medical specialties with regard to UMD and their potential clinical relevance. In addition, for UMD occurring at admission, we aimed to identify potential risk factors and evaluate the involvement of high-risk drugs.

## Methods

### Study setting and patients

This single-center, retrospective study was conducted at seven reference wards for the introduction of the unit-dose dispensing system at a tertiary care hospital in Germany. Reference wards representing different medical specialties were included, and all eligible adult patients receiving oral medication during the predefined study days were assessed. Medical specialties of the wards were urology (1 ward), cardiology (1), orthopedic surgery/orthopedics (1), psychiatry (2), mixed surgery (1) and mixed cardiology/gastroenterology (1). These wards were selected to reflect in a cross-sectional study sample of routine clinical practice across different disciplines, including medical and surgical specialties, with varying patient characteristics, medication burdens, and therapeutic focuses. Eligible patients were aged ≥ 18 years and had undergone an internal dispensing error evaluation by the hospital pharmacy in November 2023. On each of the seven wards and on two different days, oral medication prepared in the patient individual dispenser by nursing staff was compared to the prescription in the electronic prescribing software Meona® (CPOE-CDSS; Mesalvo GmbH, Freiburg, Germany, version number 2024.4.13). In this context, the preparation of medication by nursing staff in patient-specific dispensers is referred to as “dispensing” and does not include medication administration. As some patients were present on both study days, they were assessed twice in the dispensing error evaluation. However, these patients were only evaluated once at admission, ward transfer, and at discharge. Patients with complete data were included in the study cohort.

### Data collection

Medication and clinical data were collected from Meona® for regular wards and the electronic prescribing system QCare® (Health Information Management GmbH, Bad Homburg, Germany, version number 5.0.187.3) for intensive care units. Data cannot be transferred electronically between systems. Laboratory values as well as admission and discharge letters were extracted from the clinical information system (SAP i.s.h.med, Cerner Corporation, North Kansas City, USA). For all included patients, medications and all changes were assessed at defined time points starting from hospital admission until discharge. First, the entry of in-hospital medication was compared to the prehospital medication. To assess the best possible medication history, a structured review of multiple information sources routinely documented at hospital admission in the clinical information system was performed (e.g. diagnoses and documented chronic therapies, records from prior hospitalizations, medication lists from previous admissions, medication lists provided by patients (self-written or from general practitioner), medication lists documented during anesthesia preoperative assessment, transfer and discharge summaries). When discrepancies could not be resolved or the pre-admission medication remained unclear despite review of these multiple sources, the patient was excluded from the study. On three of the seven reference wards (urology, orthopedic surgery/orthopedics and mixed surgery) pharmacists were responsible for taking drug history and suggestion of in-hospital medication, which is checked, adjusted if necessary and prescribed by the attending physician. Dispensed oral medication was compared to prescriptions in Meona® as described above. At change of ward (between different regular wards or to/from ICU), prescribed medication was compared between the previous and new ward. At discharge, the discharge letter was compared to the final day’s medication record documented in Meona®. A discharge letter is a mandatory written discharge summary including information on discharge medication and recommendations on further medication therapy which has to be handed to the patient and general practitioner. The general practitioner is expected to prescribe the appropriate drug therapy based on this information. For all drugs, strength, administration form and frequency or pausing was documented. Changes at the defined assessment points were retrospectively classified as intended or unintended (UMD) by two experienced pharmacists with postgraduate training in clinical pharmacy, and several years of experience in hospital pharmacy practice and medication reconciliation. If no consensus was reached or a drug was classified as self-medication, the changes were classified as intended. Each UMD was counted only once at the specific medication process step at which it was identified, and discrepancies were not double-counted or carried forward across subsequent process steps.

### Primary and secondary outcomes

The primary outcome parameter were technical UMDs occurring during the hospital stay evaluated at admission, in-hospital transfer, oral medication dispensing and discharge. These discrepancies were independently assessed by two clinical pharmacists and classified according to their potential clinical relevance (minor, moderate, or severe) [adapted from Cornish 2005] and, if possible, changes in clinical parameters associated with UMDs (e.g. missing antihypertensive and rise in blood pressure). The assessment of clinical changes associated with UMDs was based on clinical judgment by two clinical pharmacists, taking into account the individual patient context (including information documented in medical records, e.g. clinical notes), underlying conditions, baseline clinical values, and the expected clinical effect of the discrepancy. No predefined numerical cut-off values were applied, to reflect clinical practice, where fixed thresholds may not adequately capture patient-specific relevance. Discrepancies were classified using an adjusted Medication Discrepancy Taxonomy [4]. At admission, high-risk drugs were categorized based on two published lists [28, 29]. Additional secondary outcomes included the medications involved in UMDs according to the Anatomical-Therapeutic-Chemical (ATC) code [30] and the persistence of discrepancies throughout the medication process. Examples illustrating the classification of UMDs are provided in the Additional File 1.

### Statistics

Descriptive statistics were performed with SPSS 2022 (IBM Corp., Armonk, NY, USA). Qualitative variables are expressed with their frequency distribution, while quantitative variables are presented as medians and interquartile ranges (IQR) for non-normally distributed data. For comparison between groups, the Mann-Whitney U test (non-normally distributed data) was used. Statistical significance was accepted at *p* < 0.05. A multivariable logistic regression analysis was conducted to identify risk factors associated with the dependent variable “occurrence of at least one UMD” in the admission process. Ward-level variables were not included, as the analysis was performed at the patient level. Independent variables were selected based on a literature review [11, 31–34] of similar regression models and on their availability within the retrospective study design. Variables were included in the multivariable analysis if they met the criterion of *p* < 0.1 in univariable logistic regression. The following variables were tested: age (metric: years), sex (categorical: female (reference), male), glomerular filtration rate (eGFR CKD-EPI 2009; metric: ml/min/1.73 m^2^), international normalized ratio (INR, metric), serum sodium (metric: mmol/l), serum potassium (metric: mmol/l), number of drugs at admission (categorical: 0–4 (reference), 5–9, ≥10), professional group conducting the medication history (categorical: ward staff (reference), pharmacist, ward staff followed by pharmacist), and pre-hospital stay (categorical: home-elective (reference), home-emergency, nursing or long-term care facility). The analysis was conducted using the forward stepwise regression (Wald method) and was verified using the Enter method.

During the preparation of this work the author(s) used OpenAI ChatGPT (GPT-5) (San Francisco, CA, USA) to improve readability and language. The text was reviewed afterwards, and the authors take full responsibility for the content of the publication.

## Results

### Patient cohort and UMDs

**Fig. 1:**
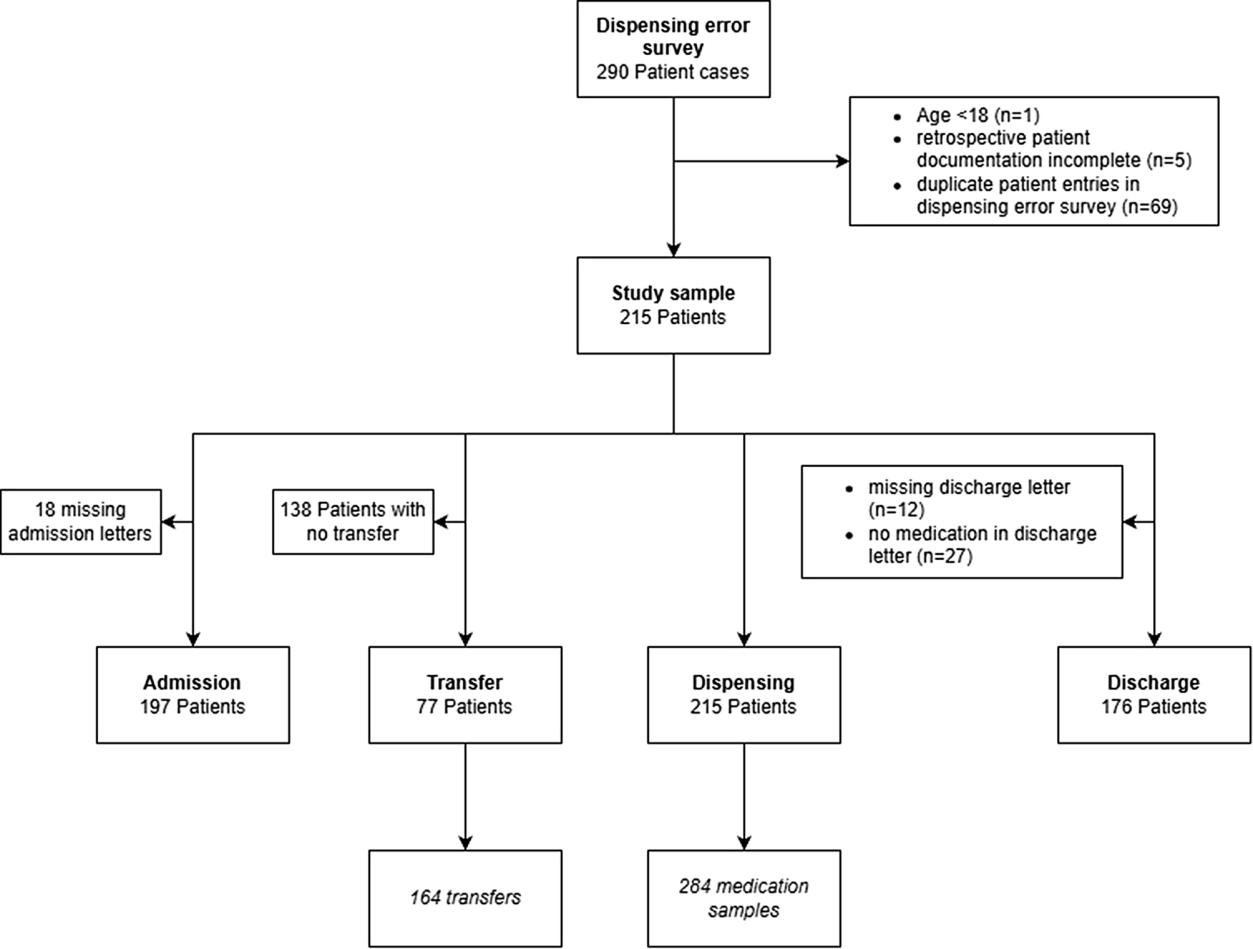
Study flow diagram

A total of 290 patient cases were evaluated for inclusion on the predefined study days in November 2023, of whom 215 patients were included in the final analysis. Figure 1 shows the patient flow chart from study inclusion to evaluations at the different time points.

Table 1 shows the patient characteristics at admission, wards of the dispensing error survey and the number of drugs at the different assessment points in the medication process.

**Table 1:** Patient characteristics at admission ward, dispensing error survey distribution, and drug numbers across assessments (*n* = 215)

**Patient characteristics at admission**		
Age (years)	71 (57.5–81.5)	
Female sex	99 (46%)	
Length of stay (days)	11 (6–30)	
Serum-Creatinine (mg/dl)	0.9 (0.8–1.2)	
eGFR (ml/min/1.73 m^2^) CKD-EPI 2009	75 (54–90)	
International Normalized Ratio (INR)	1.1 (1.0–1.2)	
Serum Sodium (mmol/l)	139 (137–141)	
Serum Potassium (mmol/l)	4.3 (4.0–4.7)	
**Number of patients by type of ward**		
Urology	30 (14%)	
Cardiology	26 (12.1%)	
Orthopedic surgery/orthopedics	27 (12.6%)	
Psychiatry	39 (18.1%)	
Mixed Surgery	54 (25.1%)	
Mixed Cardiology/Gastroenterology	39 (18.1%)	
Number of drugs per patient at assessment points*		
Admission	7 (3.0–10.5)	
Transfer (regular ward)	12 (9.0–16.0)	
Transfer (intensive care unit)	19.5 (13.0–25.0)	
Dispensing	9 (6.0–14.0)	
Discharge	8 (5.0–12.0)	

Numbers are expressed as n (%) or as median (IQR); *number of patients varying, see Fig. 1; Assessment points refer to predefined steps of the medication process at which evaluations were performed (admission, in-hospital transfer, medication dispensing and discharge)

**Fig. 2:**
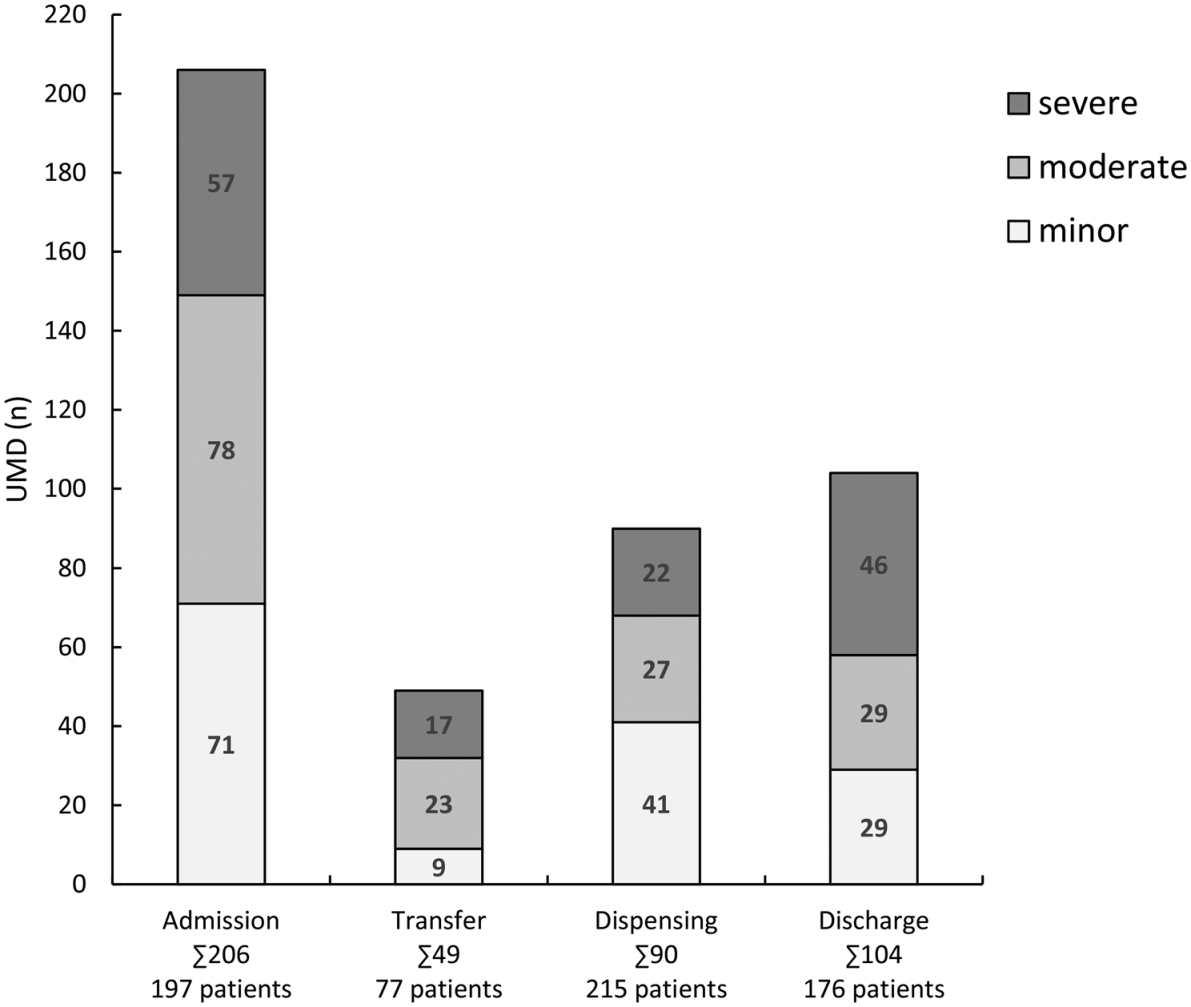
Number and severity of UMDs at each assessment point

Throughout the entire medication process, 149 (69.3%) of 215 patients experienced at least one UMD, resulting in 449 UMDs (Median 1 per patient, IQR 0–3, Range 0–15). The number and severity of UMDs at each assessment point are shown in Figure 2 and further characterized in the following paragraphs.

### UMDs at admission

On admission, 92 (46.7%) of 197 patients had at least 1 UMD (Median 0, IQR 0–2) with a wide range of 0–10 UMDs. The most common type of the 206 overall admission UMDs was omitted medications (*n* = 120, 58.3%), followed by dosing errors (*n* = 32, 15.5%) and incorrect strengths (*n* = 27, 13.1%). For 37 UMDs, an association with changes in clinical parameters was found. These included high/low blood pressure episodes following missing or wrong antihypertensive drugs (*n* = 9, 24.3%), decreased oxygen saturation following missing or wrong drugs for obstructive airway diseases (*n* = 9, 24.3%) and hyper/hypoglycemia following UMDs in antidiabetic medication (*n* = 7, 18.9%). In 53 UMDs (25.7%), the medications involved were classified as high-risk drugs. The most frequent type of discrepancy among these was omission (*n* = 34, 64.2%). The most commonly involved medications were lorazepam (*n* = 5, 9.4%), as well as buprenorphine, edoxaban, and metamizole (each *n* = 3, 5.7%).

### UMDs during ward transfers

During transfers between wards, 23 (29.9%) of 77 patients experienced at least one UMD (Median 0, IQR 0–1, Range 0–6). In total, four UMDs occurred during 92 transfers between general wards, while 45 UMDs were identified during 72 transfers to or from ICU. The number of discrepancies was significantly lower in general ward transfers compered to ICU-related transfers (*p* < 0.001). As in the admission process, the most common type of discrepancy was omitted medications (*n* = 30, 66.7%) followed by additional medications (*n* = 6, 13.3%) and incorrect dosage forms (*n* = 5, 11.1%). Clinical parameter changes associated with UMDs were observed with 9 UMDs (only in ICU transfers), most often constipation (missing laxative; *n* = 3), high blood pressure episodes (missing antihypertensive drugs; *n* = 2), and insomnia (missing hypnotic; *n* = 2).

### UMDs in dispensing

At dispensing of oral medication, 51 of 215 patients (23.7%) were found to have a least one UMD (Median 0, IQR 0–0, Range 0–5) out of 2866 dispensed drugs. The number and type of UMDs in dispensing are presented in Table 2. Out of 90 UMDs, dispensing of an incorrect single dose was the most often found error (*n* = 24).

**Table 2:** Number (%) and type of UMDs in the dispensing process (*n* = 90)

Type of UMDs	n (%)
Incorrect single dose	24 (26.7)
Incorrect tablet splitting	17 (18.9)
Documentation error	16 (17.8)
Incorrect strength	10 (11.1)
False drug (identity)	10 (11.1)
Incorrect administration time	6 (6.7)
Incorrect dosage form	5 (5.6)
Assignment to prescription not possible	2 (2.2)

### UMDs in discharge letters

In the discharge letters, 104 UMDs were identified with 60 out of 176 patients (34.1%) showing at least one UMD (Median 0, IQR 0–1, Range 0–10). As in the admission, omitted medications represented the most frequently found type of discrepancy (*n* = 83, 79.8%) followed by commission errors (*n* = 8, 7.8%), wrong frequency (*n* = 7, 6.7%) and wrong strength and pharmaceutical form (each *n* = 3, 2.9%). Among the 104 identified UMDs, 50.5% occurred at the time of admission and persisted during the hospital stay.

### Drugs involved in UMDs

**Fig. 3:**
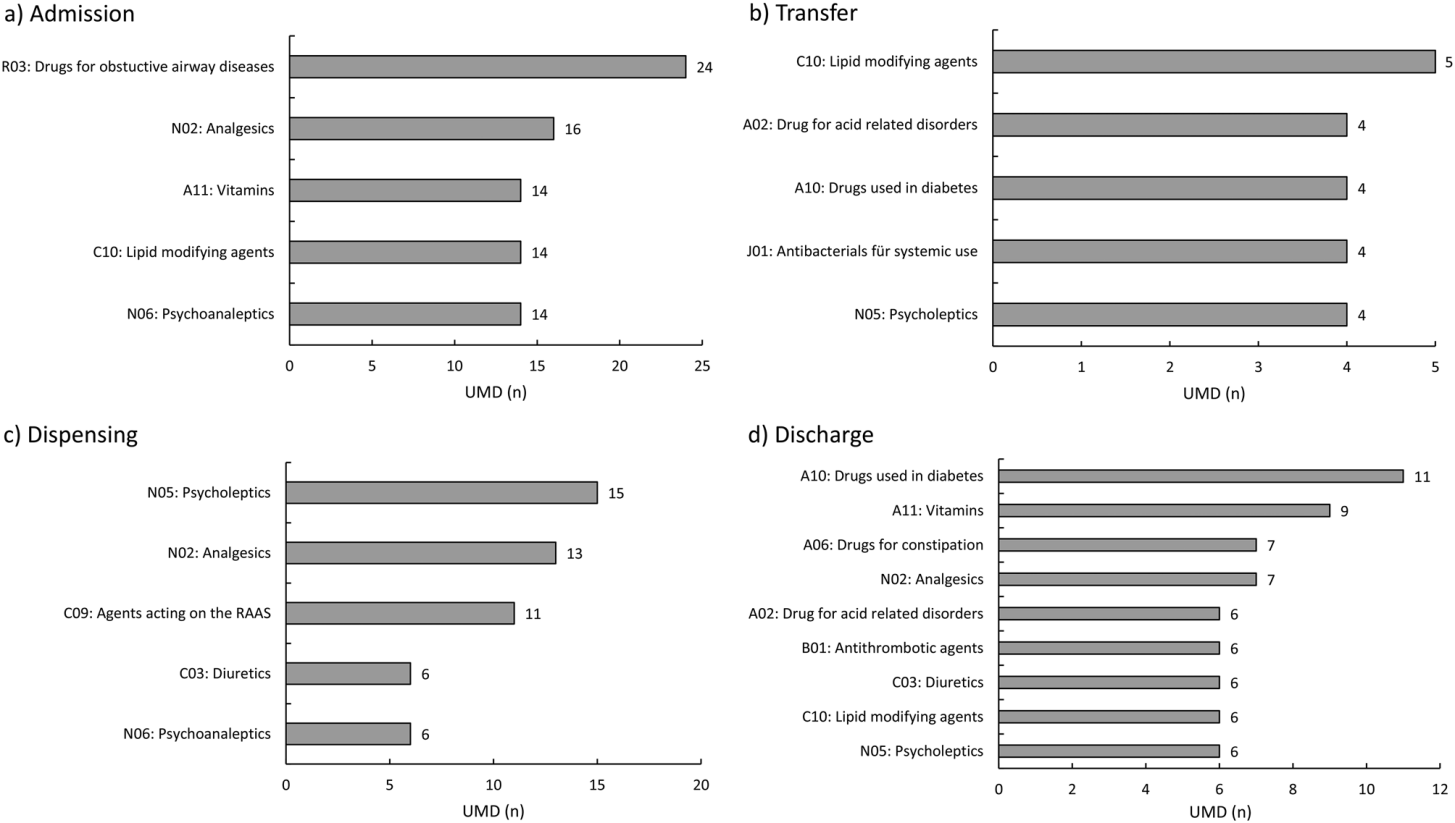
TOP 5 medication groups involved in UMDs categorized by Anatomical-Therapeutic-Chemical (ATC) code

The specific drugs involved in UMDs at the different assessment points are shown in Fig. 3. The most frequently found ATC-group related to UMDs in admission were drugs for obstructive airway diseases, exclusively inhalers, while antidiabetic drugs, especially insulins, were responsible for most of the UMDs in discharge letters. Dispensing UMDs of oral medication often involved psycholeptics, especially quetiapine.

### Factors associated with admission UMDs

Due to the highest number of UMDs being found during the admission process, we performed a logistic regression analysis to examine probable risk factors for the occurrence of UMDs at admission. The variables ‘sex’ and ‘serum potassium’ were excluded following the univariable regression. The multivariable logistic regression model showed an acceptable variance explanation (R^2^ = 0.283, *p* < 0.001). Fourteen cases were excluded from the analysis due to missing data in one or more variables. Correlations between the predictors were all low (*r* < 0.6), indicating that multicollinearity did not confound the analysis [35]. Table 3 summarizes the results of the logistic regression analysis. Patients receiving ≥ 10 drugs had a more than fourfold increased risk of UMDs compared to those with 0–4 drugs. In contrast, when a pharmacist obtained the medication history, the odds of UMDs were reduced by approximately 87% compared toward staff.

The initial medication history documented by ward staff, afterwards revised by a pharmacist, indicated a substantial risk of UMDs in the original chart entry.

**Table 3:** Results of the multivariable logistic regression model of the admission process (*n* = 183 patients)

Variables	Odds-Ratio	95% Confidence Interval	p
≥5 - <9 drugs^1^	1.935	0.890–4.208	0.004
≥10 drugs^1^	4.049	1.767–9.277	
Medication history by pharmacist^2^	0.132	0.042–0.414	<0.001
Medication history by ward staff followed by pharmacist^2^	1.934	0.932–4.012	

1 categorical (reference: 0 - <4 drugs); 2 categorical (reference: Medication history by ward staff)

## Discussion

This retrospective study identified and analyzed UMDs throughout the entire medication process for a broad patient cohort across different specialties and levels of care. A high number of patients (69.3%) experienced at least one UMD during their hospital stay, some of which were potentially clinically significant and severe. The admission process was the most critical, with a high number of UMDs and 50.5% of UMDs from admission persisted throughout the medication process and appeared in the discharge letter. Significant risk factors for UMDs occurring at admission were ≥ 10 drugs and no involvement of pharmacists for obtaining the medication history. Furthermore, we observed a significant difference between transfers within wards where a shared CPOE-CDSS system was used and transfers between general wards and intensive care units where no interface existed between the systems.

In this study, the highest number of UMDs occurred at the time of hospital admission. The most frequent error type was omission of a medication, which is consistent with observations reported in the literature [8–11]. About one third of UMDs was classified as severe emphasizing that UMDs do not only involve less critical medications or vitamins, but also essential drugs such as antidiabetics and psycholeptics. It should be noted that severity classification and high-risk medication status represent distinct concepts. Not all severe discrepancies involved high-risk medications, and not all discrepancies involving high-risk drugs were classified as severe. Given their potential to directly impact the clinical course, patient safety, and treatment costs, such errors are of particular concern [2, 3].

In our study, admission errors were often not detected or corrected during the hospital stay, which may subsequently affect outpatient care. This finding is in line with previous studies showing that discrepancies between the admission medication list and the patient’s actual medication use can persist throughout hospitalization and may be reflected in the discharge summary [36, 37]. Missing home medications in the discharge letter can lead to discontinuity of therapy [38]. Although the median number of UMDs at admission was 0, the wide range observed indicates that a subset of patients experienced multiple discrepancies. Therefore, it is important to consider potential risk factors that may contribute to the occurrence of such discrepancies. In line with other studies, the number of medications was identified as a risk factor [33, 34]. Patients taking ≥ 5 to < 9 medications showed a trend toward a higher likelihood of experiencing a UMD at admission, whereas patients taking ≥ 10 medications were substantially more likely to experience a UMD. The odds increased (OR 1.935, 95% CI 0.890–4.208) for patients with ≥5 to < 9 medications and more than fourfold for those with ≥10 medications. Possible explanations include the increased complexity of pharmacotherapy, which complicates accurate and complete medication reconciliation, especially in older patients [39].

Consistent with other findings, pharmacist-led medication reconciliation was associated with a significantly lower occurrence of UMDs [21–23, 27]. Interestingly, there was also a higher rate of UMDs when ward staff first entered the patient’s medications into the chart, followed by a pharmacist’s review and notation of discrepancies. This may be due to physicians overlooking the pharmacist’s notes, to a prioritization of inpatient treatment over the patient’s home medication list, or to the fact that pharmacist annotations were often implemented only with delay, after an error had already occurred. This finding also suggests that the actual number of UMDs at admission may be underestimated. Given the retrospective study design, only the best available medication history documented in the records, and no additional patient interviews were performed on wards without pharmacist involvement. This may have led to incomplete or inaccurate medication histories and, consequently, to an underestimation of UMDs. Nevertheless, more UMDs were detected when a pharmacist review had been performed, which might otherwise have gone unnoticed. Overall, this highlights that while pharmacist involvement improves error detection, timely recognition of their recommendations is crucial to prevent UMDs

With regard to intra-hospital transfers, the findings highlight the importance of technical infrastructure. When the same documentation system is used across wards or specialties, medication transfer is straightforward and less error-prone. In the absence of such an interface, as was the case between intensive care units and general wards, medication transfer must be done manually via the chart or transfer letters, significantly increasing the risk of errors. However, transfer letters from ICU are often incomplete, containing incorrect or missing information, resulting in medication errors potentially leading to patient harm [40]. Although the higher rate of UMDs observed in ICU patients may be partly attributable to the greater number of medications, the magnitude of the difference in UMDs (45 vs. 4 UMDs) indicates that polypharmacy alone does not fully account for the increased risk. Our results are in line with other studies characterizing the transfer between ICU and normal ward as a risk for UMDs and medication errors [41]. As this separation of documentation systems between ICU and general wards is not universal, this finding may limit the external validity of our results with regard to ICU-to-ward transfers in institutions using fully integrated electronic systems.

The magnitude of dispensing errors observed in our survey is consistent with the findings of previous studies on this topic [14–17]. It is noteworthy that the dispensing error evaluation focused exclusively on oral medications. In this context, undetected dispensing errors may be particularly relevant, as oral solid dosage forms are typically removed from their original packaging during preparation, which may reduce the visibility of identifying information such as the medication name and strength. Once prepared, these medications are usually administered without an additional professional verification step at the time of administration, making subsequent detection less likely. In contrast, medications such as injectables and inhalers are generally prepared immediately prior to administration and remain in their original packaging with clear labeling, which may facilitate identification at the point of use. According to the literature, one of the significant causes of dispensing errors is distractions and interruptions during the preparing and administration of medications [42].

In our study, we found that the reconciliation of inpatient medications with the discharge letter was often inadequate, potentially leading to errors. This is consistent with findings from a systematic review, which revealed that following hospital discharge, over 50% of adult patients experienced medication errors or UMDs, with half of these patients suffering medication-related harm [43].

Limitations of this study are its retrospective and single-center design. Missing information, e.g. concerning admission medication or possibly undocumented clinical consequences of UMDs, could not be retrieved retrospectively. Similarly, in 27 discharge summaries, no discharge medication was listed, which prevented the identification and assessment of potential UMDs. Overall, the number of UMDs might have been underestimated for these reasons, and incomplete documentation may have introduced selection bias, as only patients with sufficiently documented data could be included in specific analyses. In addition, potential differences between wards were not explicitly adjusted for in the statistical analysis, which may have influenced the observed associations. Another limitation concerns the assessment of identified UMDs, which was performed by two clinical pharmacists. While these professionals are highly qualified and experienced in evaluating medication safety, it cannot be excluded that other healthcare professionals, such as physicians, nurses or risk managers, might have assessed certain cases differently. Therefore, the results may be subject to observer bias. In addition, clinical changes were assessed as being associated with UMDs. However, a direct causal relationship cannot be established, as clinical parameters may be influenced by multiple factors beyond medication discrepancies (e.g. underlying disease, acute clinical conditions, or stress) Furthermore, this analysis was conducted under the specific conditions of the study hospital, including its established processes, documentation standards, electronic systems and ward staff. Due to differences in institutional regulations, staff resources or interprofessional communication pathways, the transferability of the findings to other settings may be limited. The single-center design allowed for a consistent and detailed assessment of medication discrepancies within one institutional setting but may limit generalizability. Seasonal variation in patient characteristics cannot be excluded due to the limited data collection period. Furthermore, medication administration was not directly assessed. Although it can be assumed that oral medications prepared in patient-specific dispensers are generally administered as dispensed, this could not be verified within the retrospective study design. Nevertheless, we believe the overall findings will be of concern and give valuable suggestions for action to many hospitals.

Previous studies have typically focused on specific stages of the hospital medication process, with some covering more than one stage, often in selected patient cohorts [38, 44, 45]. In contrast, our study examines the full medication pathway, including admission, intra-hospital transfers, dispensing, and discharge, across a broad and heterogeneous patient cohort. This comprehensive perspective enables the identification of errors throughout the entire hospital stay, providing a more complete and generalizable understanding of medication errors than studies limited to individual or partial stages.

Our study provides a thorough evaluation of UMDs throughout the medication process. This enables UMDs to be identified at every key stage of the hospital stay. Using real-world data from routine hospital practice means our findings reflect actual clinical conditions and provide unique insights into the frequency and types of discrepancies that may impact patient safety during and after hospital stays. The results of this study are of general interest to hospitals in terms of ensuring the safety of drug therapy throughout the technical medication process. Furthermore, based on these results, additional measures will be developed and implemented alongside the introduction of the unit dose dispensing system in our hospital. Beyond this, future studies may further explore the impact of ward-level factors on the occurrence of UMDs, particularly in the admission process, in larger cohorts enabling multilevel analyses to better account for structural differences between wards and specialties.

## Conclusion

This study demonstrates that unintended discrepancies in medication can occur at any stage of the in-hospital medication process and represent a severe patient safety issue, particularly at admission. The findings emphasize the importance of improving this high-risk process to prevent patient harm and enhance drug therapy safety. Our findings highlight potential targets for future interventions aimed at improving medication safety, including process optimization, the involvement of clinical pharmacists at key steps of the medication process, and system-level approaches. In particular, structured dispensing processes and improved integration of electronic prescribing systems across departments may represent important strategies to reduce medication discrepancies

## Electronic supplementary material

Below is the link to the electronic supplementary material.

Supplementary material 1

## Data Availability

The datasets used and/or analyzed during the current study are available from the corresponding author on reasonable request

## Abbreviations

UMDs: Unintended medication discrepancies
ICU: Intensive care unit
CPOE: Computerized Physician Order Entry
CDSS: Clinical Decision Support Systems
ATC-Code: Anatomical-Therapeutic-Chemical-Code
IQR: Interquartile ranges
eGFR: Glomerular filtration rate
INR: International normalized ratio
