# Supplementary material for: Unintended medication discrepancies across key stages of the in-hospital medication process: a retrospective real-world study in hospitalized patients
Source: BMC Health Serv Res. 2026 Apr 18;26:552. doi: 10.1186/s12913-026-14581-4 (PMC13097707; doi:10.1186/s12913-026-14581-4)
Supplement: Supplementary file 1 — Supplementary material 1 [file 12913_2026_14581_MOESM1_ESM.docx]

Additional File 1: Examples of classification of unintended medication discrepancies (UMDs) according to severity [modified from Cornish 2005]

| Example of UMD |  | Severity |  |
| --- | --- | --- | --- |
|  | Minor | Moderate | Severe |
| Missing inhaler (e.g. salbutamol) | Patient uses inhaler only occasionally (<1×/week); unlikely to be clinically relevant | Patient uses inhaler as needed (>1×/week); could be clinically relevant | Patient requires regular inhalation due to underlying chronic respiratory disease (e.g. COPD or asthma), making omission clinically relevant |
| Wrong drug within the same class (e.g. atorvastatin instead of rosuvastatin) | Equivalent dosing; no additional risk factors, with no clinically relevant change or harm expected | Non-equivalent dosing without additional risk factors (e.g. normal renal function), with no immediate clinical harm expected | Non-equivalent dosing with risk factors (e.g. impaired renal function), with immediate clinical harm expected (e.g. rhabdomyolysis) |
| Tablet split incorrectly | Splitting not recommended according to product information, but clinically negligible dose deviation | Splitting not recommended; small dose deviations may be clinically relevant | Splitting not possible (e.g. modified-release formulation); risk of dose dumping |
| Medication omitted in discharge letter | As-needed medication rarely used during hospitalization | As-needed medication regularly used and likely required after discharge | Chronic medication with high likelihood of unintended harm due to discontinuation in outpatient care |
